# Ambulatory care-sensitive emergency department cases: a mixed methods approach to systemize and analyze cases in Germany

**DOI:** 10.1093/eurpub/ckz081

**Published:** 2019-05-14

**Authors:** Wiebke Schuettig, Leonie Sundmacher

**Affiliations:** Department of Health Services Management, Ludwig Maximilians University Munich, Munich, Germany

## Abstract

**Background:**

Internationally, emergency departments (ED) are treating increasing numbers of patients with conditions that could have been managed appropriately in ambulatory care (AC) settings. The aim of our study was to develop the first consensus-based list of AC-sensitive conditions commonly seen in German EDs and explore predictors of these visits.

**Methods:**

Our study used a Delphi survey of 30 physicians to compile a list of conditions they agreed were amenable to AC treatment. The group identified reasons why patients visit EDs instead of AC. We used the results to inform spatial regression models analysing the association of patient characteristics and attributes of AC with AC-sensitive ED visits based on 2015 district-level data.

**Results:**

Our study provides a list of AC-sensitive conditions based on the German ED context. Results suggest that, up to the age of 70 years, the older the patients, the less likely they seek EDs for these conditions. Results of our regression analyses suggest that AC-sensitive ED rates were significantly higher in districts with lower physician density. Patients’ urgency perception and preferences were identified as main drivers of AC-sensitive ED visits.

**Conclusion:**

Future policy measures should aim to help guide patients through the healthcare system so that they receive the best care in place that is most appropriate in terms of quality, safety and continuity of care. A list of AC-sensitive ED conditions can be used as a monitoring instrument and for further analyses of routine data to inform policy makers seeking to improve resource use and allocation.

## Introduction

In many developed healthcare systems, the number of patients seeking healthcare in emergency departments (EDs) has increased in recent decades.^1^^,^^2^ Many of these patients, however, have complaints that could be treated appropriately and often at lower cost in the ambulatory care (AC) setting.^3^^,^^4^ In many countries, an increase in such visits is one factor that has led to overcrowding in EDs.^1^ This can affect patient safety and the timeliness of treatment.^1^^,^^4^^,^^5^

In statutory health insurance (SHI) systems like that in Germany, where there is no gatekeeping to services provided by secondary care, patients can directly access GPs, specialists and EDs free at the point of use.^2^^,^^6^ After-hours services in AC are organized by regional associations of SHI physicians and range from telephone counselling to out-of-hours appointments at specific practices, as well as home visits. The planning, funding and provision of AC and hospital services in Germany are not joined up in any substantial way. This fragmentation has led to a lack of care coordination for patients across these sectors.^7^ Consequently, it is predominantly patients who decide, based on their preferences, where they will seek care.^6^ Regular appointments in AC may involve waiting times whereas ED visits promise timely and comprehensive diagnostics and treatment.^8^ Combined with perceptions of pain and urgency, health anxiety and a lack of information about how the health system works, this may contribute to a preference to attend EDs.^9^

A number of studies have attempted to systemize the classification of potentially avoidable ED visits.^10^^,^^11^ One branch of literature focuses on the prospective assessment, in EDs, of patient symptoms by medical staff or patients themselves.^12^^,^^13^ Another branch systemizes the classification of avoidable ED visits retrospectively based on diagnoses.^14^^,^^15^ For example, Billings *et al.* developed a profiling algorithm to analyze patient visits to EDs.^14^ The algorithm classifies ED use into four categories: non-emergent, emergent and primary care preventable, emergent and primary care treatable and emergent and not preventable. The algorithm was used predominantly in the US context and has not been updated since 2000.

Another related branch of literature draws on the concept of AC-sensitive conditions and analyses hospitalizations that are potentially avoidable by effective treatment of acute conditions or management of chronic conditions in the AC sector.^16–19^ The concept provides an instrument to investigate access to AC services, as well as their quality and effectiveness using potentially avoidable hospitalizations as an outcome measure.^16^ For the purposes of the present study, AC-sensitive hospitalizations and AC-sensitive conditions in EDs share the same conceptual approach to explore the potential implications of a lack of access to or limited use of AC. It should be noted that, AC-sensitive conditions in ED focus narrowly on potentially avoidable ED cases as an outcome measure. It is well understood that both hospitalizations for AC-sensitive conditions and ED cases cannot always be prevented through AC.^17^ Exogenous factors such as socioeconomics, patient preference and compliance confound the relationship between AC and hospitalizations.^18^^,^^19^

Focusing on ED cases amenable to AC the present study had the following three aims:

1. Based on group consensus methods, our first aim was to develop an instrument to systemize the identification of ED cases amenable to treatment with AC. These cases include patients treated on the same day in hospital EDs but exclude emergency admissions. Additionally, we identified potential reasons why patients seek EDs rather than AC.
2. The second aim was to describe characteristics of patients visiting EDs with AC-sensitive conditions based on German data from 2015.
3. The third aim was to investigate the relationship between patient characteristics, AC settings, and the rate of AC-sensitive ED cases using linear spatial models at the level of German districts.

## Methods

We conducted a Delphi survey among 30 German physicians to pursue the first aim of our study. The Delphi technique is an iterative multistage process designed to transform opinion into group consensus.^20^ The panellists worked in hospitals or AC and were selected based on medical disciplines and locations.

Three physicians not on the Delphi panel piloted the questionnaires before the survey to ensure that these were consistent and easy understandable. In the first round, we introduced the panellists to the concept of AC-sensitive conditions and presented them with a list of conditions consisting of most frequent ICD-10 diagnoses in EDs and after-hours services in Germany. Using an anonymous, interactive online survey, we asked the panellists to classify each condition as being (a) treatable with AC, (b) treatable with after-hours services or (c) treatable only in EDs. We invited panellists to suggest additional conditions. In cases where more than 70% of the panellists agreed that a condition was (a), we added this condition to a semi-final list.^21^ After all panellists had finished the first round, they received an email detailing their results and how these compared with those of the group.

In the second round, we presented the panellists with diagnoses that had not reached at least 70% agreement in the first round. We asked them to classify these diagnoses once more and allowed them to alter their previous decisions. For the remaining questions, we aggregated relevant ICD codes into disease groups based on their ICD chapters. Because AC-sensitive conditions cannot always be prevented/treated with AC,^17^ we asked panellists to estimate the percentage of ED cases in each group that they felt, based on their experience, were preventable with AC treatment. We also asked the panellists to choose among the following seven reasons why patients in each group might seek ED services rather than AC: need for diagnostics, patient comorbidities, patients’ preference to seek EDs rather than AC, AC physician referrals, patients’ perception of an emergency, a lack of regular physicians in patients’ proximity or of patient information about remits of actors in healthcare. We had compiled these reasons based on literature reviews^8–10^ and interviews. Panellists were able to add additional reasons. We set a threshold of at least 65% agreement for an AC-sensitive condition to be added to our final list.^22^

Based on 2015 claims data provided by the Federal Association of SHI Physicians and to address the second aim of our study, we calculated descriptive statistics for 10-year patient age groups who had used EDs for AC-sensitive conditions. ED cases were identified based on billing data, specifically claims from section 1.2 of the billing catalogue made by hospitals. These data include information on SHI patients in Germany except for residents of the states of Bremen, Hesse and Rhineland-Palatinate due to the presence of special remuneration agreements with direct billing arrangements (so-called Selektivverträge). The full AC-sensitive condition list was split into general medical diagnoses and injuries to address differences in how these conditions are treated.

For the regression analyses and to pursue the third aim of our study, cases at the level of German districts (338) were standardized directly by age and sex and reported as rates per 1000 inhabitants. Rates for the full list of AC-sensitive conditions and for the subsets of general medicine and injuries served as dependent variables. Based on a literature search and the Uscher-Pines conceptual model,^10^ we hypothesized that morbidity, patients’ previous healthcare experience, socioeconomics, culture and costs of seeking healthcare services would impact on ED use.

We calculated a patient-specific Charlson comorbidity index based on ICD-10 codes for any AC visit before an AC-sensitive ED case to control for morbidity.^23^ Furthermore, we calculated the average number of AC cases before an AC-sensitive ED case as a proxy for previous healthcare experience and knowledge about healthcare alternatives. We computed the percentage of non-German citizens to account for differences in knowledge about healthcare provision and cultural differences.

As voter turnout can indicate social capital and might demonstrate attitudes towards public services,^24–26^ we incorporated voter turnout statistics from federal elections in our analysis. Voter turnout has been shown to be positively associated with healthcare seeking behaviour such as attendance at screenings and we therefore hypothesized a negative association with ED use.^24^^,^^26^

We included district-level average household income to control for differences in ED services use related to socioeconomics. Previous studies have reported differences in ED use being related to patient income^10^^,^^27^ and variations in patients’ perceptions of the urgency and availability of physicians being related to socioeconomics.^28^

The average driving minutes to the next hospital per district were included to represent the cost of seeking an ED. Additionally, we calculated physician density as the number of AC physicians per 100 000 inhabitants. This can be seen as a proxy for the availability of practices and for waiting times for an appointment in AC. The findings of previous studies suggest that convenience factors such as travel, timing and location play a major role in the utilization of EDs.^10^ Finally, we included a dummy variable for the German states in order to control for different after-hours policies and payment schemes in AC.

Data on AC-sensitive ED cases, AC cases and diagnoses were provided by the Federal Association of SHI Physicians. District-level data on household income (2014), voter turnout (2014), the proportion of non-German citizens (2014), physician density (2014) and travel time to hospitals (2015) were calculated using data from the Federal Office for Building and Regional Planning (BBR).

We assessed the association between personal characteristics and characteristics of AC and ED rates using linear regression models. First, linear regression models via OLS were estimated and tested for spatial dependence in the residuals using Moran’s I statistic. We assumed spatial autocorrelation resulting from unobserved spatial factors or spatial error correlation. Examples of such spatial factors are unobserved regional clusters of morbidity or unobserved cultural differences among regions in the use of EDs. By means of a Lagrange multiplier test, we tested for unobserved spatial influence on the rate of AC-sensitive ED cases (spatial error model).

We defined the weight matrix as a contiguity matrix indicating connections between districts, and we operationalized connections between districts by defining a distance in driving minutes of fewer than 75 min. Driving minutes were provided by the BBR.

We fitted spatial error models using maximum likelihood estimation techniques. The software package STATA 13 was used to run analyses.

## Results

The pilot study confirmed the feasibility of the questionnaire. In the first Delphi round, 152 ICD-10 codes were evaluated by the panellists (see table 1). They proposed an additional 82 codes to be included in the list. In the second round, 135 conditions were identified at an agreement level of 65% or higher as being AC-sensitive. These were aggregated to the 31 disease groups shown in table 2. Answers on whether conditions were treatable with after-hours services varied considerable across disease groups, with lower rates assessed by hospital than by AC physicians. Acute nasopharyngitis, for instance, was assessed by 100% of the panellists as treatable with AC. In contrast, 42% assessed the disease as being treatable through after-hours services (55% ambulatory, 31% hospital representatives). The average level of preventability was 70%.

**Table 1:** Structure and results of the Delphi survey

Selection and invitation of panellists			Round 1 of the Delphi survey	Round 2 of the Delphi survey
**Selection of panellists:** Criteria: Ambulatory care sensitive relevant medical disciplines, representative of ambulatory/hospital sector and location			**Selection of proposed diagnoses:** List of 100 most frequently coded diagnoses in EDs and after-hours services in 2013 reduced by duplicates. Identification of 152 ICD-10 codes **Structure of the online questionnaire:** Presentation of detailed background information on the concept of ambulatory care-sensitive conditions Assessment whether an ED case with a respective diagnoses was also treatable by ambulatory care/after-hours services/only treatable in ED setting. Treatment out-of-office was explained as a subset of ambulatory care Participants were invited to suggest additional diagnoses. Participants were allowed to choose multiple answers and to skip questions ICD codes were displayed in hierarchical order with ICD codes and description **Main results:** Thirty physicians completed the first round. Eleven ICD codes received at least 70% agreement of being treatable by ambulatory care and after-hours services → semi-final list Forty-nine ICD codes were evaluated by at least 70% as treatable by ambulatory care → semi-final list Seven diagnoses with at least 70% participants voting the diagnoses as only treatable by EDs ICD codes which did not get at least 70% consensus for one answer were again proposed in round 2 Eighty-two additional ICD codes (three and four-digit codes) were suggested by the participants All physicians that completed round one received an individual feedback sheet via email with the individual results in relation to the results of the group **Timeframe of the first round**: March 2015–August 2015	**Selection of proposed diagnoses:** List of ICD codes that did not receive 70% acceptance for one answer category in the first round List of ICD codes additionally suggested by participants **Structure of the online questionnaire:** Presentation of detailed background information. Validation whether emergency cases with a respective diagnosis was treatable by ambulatory care/after-hours services/only treatable in ED setting Assessment of the degree of preventability of the treatment in EDs of a disease group on a scale from 0 to 100% Subjective assessment of reasons for patients seeking EDs for individual disease groups with proposed reasons: (1) the need for diagnostics to exclude suspected diagnoses, (2) patient comorbidities, (3) patients’ preference to seek EDs rather than regular ambulatory care, (4) referrals of ambulatory care physicians, (5) patients’ perception of an emergency, (6) a lack of regular physicians in patients’ proximity, (7) lack of patient information about the remit of different actors in the health sector **Main results:** Twenty-five physicians completed the second round. One hundred thirty-five ICD codes received at least 65% agreement of being treatable by ambulatory care and were considered as AC sensitive. An average of 70% of the AC-sensitive disease groups were assessed as preventable Most often chosen answer regarding the reasons why patients rather visit ED than regular care was (5) with 194 votes, followed by (3) with 116 **Timeframe of the second round**: August 2015–December 2015
**Invitation of panellists:** Invitation sent via email or mail by the study group to potential candidates with information about the study setting.				
**Recruited panellists:**				
*Specialized field*	*Ambulatory care*	*Hospital care*		
General medicine	5			
Internist		5		
Anesthesiology	1	2		
Emergency medicine, trauma surgery		3		
Internist, emergency medicine	1	2		
Orthopaedics		1		
Neurology	1			
Surgeon	3	1		
ENT physician	1			
Urologist	1			
Gynaecology	1			
Trained nurse		1		
Dentist	1			
The average age of the panellists was 51 years (ranging from 31 to 66). About one third of the panellists were qualified in emergency medicine.				

**Table 2:** List of AC-sensitive diagnoses

Ambulatory care-sensitive disease group	Percentage treatable with AC/treatable with after-hours services/only treatable in EDs	Percentage of predicted preventability with AC^a^	Most often selected reason for visiting EDs instead of regular care^b^
1. Persons with potential health hazards related to communicable diseases	95%/26%/5%	74% (77%)	Preference
2. Dermatitis, eczema and other disease of the skin	93%/46%/3%	86% (83%)	Perception
3. Biomechanical lesions	91%/48%/4%	76% (73%)	Preference
4. Bronchitis	91%/70%/0%	76% (77%)	Perception
5. Persons encountering health services for examination and investigation	91%/46%/6%	87% (94%)	Preference
6. Soft tissue disorders	91%/51%/3%	73% (67%)	Perception
7. Hypertensive diseases	90%/39%/8%	72% (61%)	Perception
8. Arthropathies	89%/50%/2%	81% (80%)	Preference
9. ENT infections	88%/56%/3%	78% (81%)	Perception
10. Diseases of the eye and adnexa	88%/67%/4%	74% (70%)	Perception
11. Certain infectious and parasitic diseases	87%/46%/8%	69% (62%)	Perception
12. Back pain	87%/53%/7%	75% (68%)	Perception
13. Diseases of the digestive system	87%/54%/6%	67% (64%)	Perception
14. Diabetes mellitus	87%/43%/9%	70% (66%)	Comorbidity
15. Diseases and symptoms of urinary system	87%/57%/6%	66% (59%)	Perception
16. Persons encountering health services for specific procedures and health care	87%/45%/14%	76% (76%)	Preference
17. General symptoms and signs	83%/67%/7%	75% (72%)	Perception
18. Diseases of oral cavity, salivary glands and jaws	81%/43%/7%	80% (82%)	Perception
19. Diseases of the ear and mastoid process	80%/38%/16%	78% (75%)	Perception
20. Effects of external causes	77%/45%/27%	55% (43%)	Perception
21. Intestinal infectious diseases	77%/54%/21%	68% (63%)	Perception
22. Diseases of the circulatory system	76%/40%/18%	58% (52%)	Perception
23. Superficial injuries	76%/54%/15%	69% (61%)	Perception
24. Mood [affective] disorders	75%/32%/18%	62% (69%)	Scarcity
25. Symptoms and signs involving the circulatory and respiratory systems	73%/55%/18%	59% (46%)	Perception
26. Diseases of male genital organs	73%/40%/23%	73% (67%)	Perception
27. Diseases of the genitourinary system	73%/41%/30%	64% (56%)	Perception
28. Diseases of the nervous system	71%/36%/20%	59% (48%)	Perception
29. Contusion, sprain and strain	69%/48%/20%	67% (61%)	Perception
30. Fractures and injuries	68%/36%/28%	29% (24%)	Diagnostics
31. Infections of the skin and subcutaneous tissue	68%/51%/31%	78% (73%)	Perception

**ICD codes of disease groups: 1**: Z23^a^ Z24.4^a^ Z24.5^a^ Z24.6^a^ Z25.8^a^ Z27.4^a^ Z29.8^a^; **2**: L20.8^a^ L20.9^a^ L22^a^ L23.2^a^ L23.4^a^ R21^a^; **3**: M99.8^b^; **4**: J40^a^; **5**: Z72.8^a^ Z74.8^a^ Z75.2^a^ Z76.8^a^ Z01^a^ Z09^a^; **6**: M62.6^b^ M79.0^b^ M79.2^b^ M79.6^b^; **7**: I10.0^a^ I10.9^a^; **8**: M10.0^a^ M25.5^b^; **9**: H66.9^a^ J00^a^ J02.9^a^ J03.9^a^ J04.0^a^ J06.8^a^ J06.9^a^ J20.9^a^; **10**: H10.9^a^; **11**: B34.9^a^ B85.0^a^ B86^a^ B99^a^; **12**: M54.1^b^ M54.2^b^ M54.4^b^ M54.5^b^ M54.6^b^ M54.8^b^ M54.9^b^; **13**: K52.9^a^ K59.0^a^ K59.1^a^; **14**: E11^a^; **15**: N30.0^b^ N39.0^a^ R30.0^a^ R32^a^; **16**: Z41.9^a^ Z43.0^a^ Z43.2^a^ Z43.3^a^ Z47.8^b^ Z47.9^b^ Z48.0^b^ Z48.8^b^ Z48.9^b^ Z59^a^ Z63^a^; **17**: R50^a^ R51^a^ R52.9^b^ R53^a^; **18**: K11.2^a^ K12.0^a^ K14.0^a^; **19**: H91.2^a^ H93.1^a^; **20**: T67.6^b^ T75.2^b^ T78.4^a^ T83.0^a^; **21**: A09.0^a^ A09.9^a^; **22**: I80^b^ I83^a^; **23**: S00.0^a^ S00.03^a^ S00.3^a^ S00.8^a^ S00.9^a^ S30.0^a^ S40.0^b^ S50.0^a^ S50.1^a^ S50.8^a^ S60.0^b^ S60.2^b^ S60.8^b^ S70.0^a^ S80.0^b^ S80.1^b^ S80.8^b^ S90.1^b^ S90.3^b^ T00.9^a^ T14.0^b^ T14.03^b^; **24**: F32^a^ F33^a^ F34^a^ F38^a^ F39^a^; **25**: R04.0^a^ R05^a^ R06.4^a^ R10.1^a^ R10.3^a^ R10.4^a^ R11^a^; **26**: N41.1^a^ N45.9^a^ N48.1^a^; **27**: N12^b^ N28.8^a^; **28**: G35.1^a^ G58.0^a^; **29**: S20.2^b^ S63.5^b^ S63.6^b^ S83.6^b^ S93.4^b^ S93.6^b^, **30**: S92.5^b^; **31**: A46^a^ L03.0^b^ (a, general medical diagnoses; b, injuries).

a Percentage value of predicted preventability of panelists practicing in hospitals. b:diagnostics: the need for diagnostics to exclude suspected diagnoses, comorbidity: patient comorbidities, preference: patients’ preference to seek EDs rather than regular ambulatory care, referral: referrals of AC physicians, perception: patients’ perception of an emergency, scarcity: a lack of regular physicians in patients’ proximity, information: lack of patient information about the remit of different actors in the health sector.

The panellists most often chose patients’ perception of an emergency as cause of using ED services, followed by patient preference to visit EDs (table 2). Physicians felt that the availability of physicians facilitated ED visits particularly for diseases of the skin and psychological disorders.

In 2015, there were ∼4.4 million cases in which patients presented at German EDs with an AC-sensitive condition. This amounted to 49% of all emergency cases in the study region. The coding of ED diagnoses in Germany does not specify primary or secondary diagnosis. Because we counted cases when one of the relevant diagnoses was coded, our findings may overestimate the number of cases. Table 3 shows the gender- and age-specific distribution of AC-sensitive ED cases, morbidity and the mean number of prior AC cases per AC-sensitive ED case. For both genders, we found the highest rates of AC-sensitive ED cases in the first years of life and decreasing rates up to, but not including, the age group of 70 and above.

**Table 3:** Gender and age-specific distribution of AC-sensitive ED cases^a^

Age group	Female				Male			
	AC-sensitive ED cases per 1000	Average value Charlson Index	Mean number of diagnoses per AC-sensitive ED case	Mean number of ambulatory care cases per year	AC-sensitive ED cases per 1000	Average value Charlson Index	Mean number of diagnoses per AC-sensitive ED case	Mean number of ambulatory care cases per year
0-9	119.47	0.24	1.33	4.89	142.12	0.32	1.35	5.15
10-19	98.17	0.27	1.29	6.29	95.08	0.28	1.29	4.40
20-29	99.58	0.34	1.43	7.38	83.29	0.28	1.34	4.05
30-39	69.48	0.47	1.42	7.66	59.16	0.41	1.34	4.48
40-49	49.04	0.80	1.36	8.25	43.53	0.79	1.38	5.48
50-59	42.73	1.40	1.39	9.57	35.58	1.57	1.44	6.84
60-69	38.64	2.48	1.47	10.77	33.60	3.26	1.52	8.92
70-79	48.06	3.84	1.57	11.74	43.60	5.27	1.61	11.12
>79	76.94	4.42	1.78	9.28	76.29	5.64	1.79	10.30
Mean	71.35	1.58	1.45	8.43	68.03	1.98	1.45	6.75

a Data based on SHI population excluding Bremen, Hesse and Rhineland-Palatinate.

The positive and significant Moran’s I in models using the full AC-sensitive condition list, the general medicine only list and the injuries only list indicates a positive correlation between rates of AC-sensitive ED cases in neighbouring districts. The hypothesis that unobserved spatial variables influence these rates could not be rejected by the Lagrange Multiplier test in all models. Table 4 summarizes the results of the OLS and the spatial error models. Test statistics suggest, however, that there is no significant difference in explanatory power between the OLS and SEM models. The following interpretations refer to these results.

**Table 4:** Regression results

	AC-sensitive ED case rate		AC-sensitive ED case rate—general medicine		AC-sensitive ED case rate—injuries	
	OLS	SEM	OLS	SEM	OLS	SEM
Charlson index	0.0128^***^	0.0146^***^	0.00844^***^	0.00879^***^	0.00547^**^	0.00743^***^
	(0.00375)	(0.00370)	(0.00248)	(0.00245)	(0.00207)	(0.00201)
AC cases	−0.00569^**^	−0.00549^**^	−0.00374^**^	−0.00365^**^	−0.00262^**^	−0.00270^**^
	(0.00179)	(0.00175)	(0.00119)	(0.00116)	(0.000987)	(0.000947)
Income	−0.00448	−0.00477	−0.00320	−0.00329	−0.00162	−0.00190
	(0.00354)	(0.00338)	(0.00234)	(0.00227)	(0.00195)	(0.00181)
Voter turnout	−1.092^***^	−1.040^***^	−0.602^***^	−0.599^***^	−0.569^***^	−0.478^***^
	(0.184)	(0.180)	(0.122)	(0.119)	(0.101)	(0.0989)
Distance to hospitals	−0.220	−0.230	−0.130	−0.130	−0.114	−0.137
	(0.173)	(0.167)	(0.114)	(0.111)	(0.0953)	(0.0903)
Physician density	−0.0422^**^	−0.0422^***^	−0.0186*	−0.0186*	−0.0267^***^	−0.0271^***^
	(0.0133)	(0.0127)	(0.00877)	(0.00851)	(0.00731)	(0.00686)
% of non-German citizens	0.548*	0.514*	0.776^***^	0.765^***^	−0.0853	−0.107
	(0.217)	(0.219)	(0.144)	(0.142)	(0.120)	(0.121)
Constant	139.5^***^	132.3^***^	77.83^***^	76.67^***^	73.97^***^	66.00^***^
	(14.73)	(14.68)	(9.742)	(9.598)	(8.111)	(8.068)
λ		0.302*		0.120		0.480^***^
Observations	338	338	338	338	338	338
*R* ^2^	0.570	0.568^a^	0.531	0.529^a^	0.563	0.545^a^
Adjusted *R*^2^	0.544		0.503		0.537	
*AIC*	2506.2	2504.9	2226.8	2230.1	2103.0	2091.9
*F*	22.17		18.92		21.59	
Moran’s *I*	4.108^***^		2.456^**^		6.001^***^	
Lagrange multiplier test (spatial error)	4.809^**^		0.658		14.263^***^	

Standard errors in parentheses. Values for the individual states of Germany are displayed in the Supplementary Appendix.

a For the spatial error models a pseudo *R*^2^ equal to the ratio of the variance of the predicted values to the observed values is reported.^38^

* *P *< 0.05.

** *P *< 0.01.

*** *P *< 0.001.

We found that characteristics of AC significantly interfered in all models with the rates of AC-sensitive ED cases in any given district: Physician density and mean number of prior AC cases were negatively associated with these rates. The results suggest that an additional physician per 100 000 inhabitants is associated with a decrease of four AC-sensitive ED cases per 1000 inhabitants.

Patient-related factors were also associated with AC-sensitive ED cases: the control variable for patient morbidity was significantly positively associated with these rates in all models. Consistent with the literature, household income was negatively associated with ED cases^27^^,^^29^ but not significantly. Voter turnout was negatively associated with these rates. In districts with high voter turnout we observe fewer AC-sensitive cases.

The percentage of non-German citizens was positively associated with the rate of AC-sensitive ED cases when considering either the full or general medicine diagnosis list. For the subset of injuries, however, the coefficient was negative and not significant.

## Discussion

Following a mixed methods approach, this paper contributes to the literature on the classification and analysis of potentially avoidable visits to EDs. Using a Delphi process with a panel of 30 physicians, we provide the first list of AC-sensitive conditions common in EDs in Germany. The list can be used to compute the rate of AC-sensitive ED visits among regions in Germany, and potentially in other countries, in order to monitor use of EDs and access to AC. One important strength of the compiled list is that it can be computed using data collected routinely. Results are therefore easy to compile, comparable among regions and hospitals and not biased by self-reported urgency. Using a structured, consensus-based process that drew upon the expert opinion of a panel of physicians, we account in our list for the fact that not all of these conditions are fully preventable through AC treatment. Incorporating this fact, our results compare well with national and international studies which estimate that between 20 and 43% of such cases are treatable by AC.^30^^,^^31^

Our study provides evidence that continuity of care in AC and ease of access to AC providers may predict whether patients may seek care in EDs even if they could receive appropriate treatment in AC. In our sample, patients were significantly less likely to seek care in EDs if they belonged to an age groups with a higher average number of AC cases per year. Up to age of 70, the older our patients were, the less likely they were to seek care in EDs if they had an AC-sensitive condition. In the literature, there is evidence that patients in continuous care are less likely to visit EDs in general. Assuming that a higher average number of AC cases per year is a proxy for continuous care, this may be one explanation for our findings.^4^^,^^32^^,^^33^ Our results indicate that strengthening continuity of care and improving access to AC can help to reduce emergency cases.

We found that a higher proportion of non-German residents in a district was associated with a higher rate of AC-sensitive ED cases, especially for general conditions. While this might be due to culturally different patterns in health services use, other explanations include a potential lack of familiarity with the health system^34^ and language barriers. We also found that the proportion of AC-sensitive cases treated in EDs was significantly higher in areas with low voter turnout. If we interpret voter turnout as a proxy for social participation and capital, this finding might underscore the idea that better patient information and healthcare navigation could lead to more appropriate care provision. Indeed, in our Delphi survey, physicians selected ‘patient preference’ and ‘patient perception’ as the prime explanations for why patients seek ED rather than AC.

This study has a number of important limitations. First, our Delphi approach did not provide an exhaustive list of AC-sensitive conditions. Rather, it is based on the consensus of 30 physicians and should be seen as a preliminary tool to classify ED visits in future research a more systematic way. Second, the credibility of any consensus method depends in large part on the composition of the participating panel.^35^ We attempted to generate a valid indicator by involving a diverse panel of physicians comprised of equal numbers from the AC and hospital settings (table 1), as well as from a broad range of medical disciplines and regions across Germany. The results must nevertheless be regarded as influenced by the physicians’ personal experience and medical knowledge. Third, the concept of AC-sensitive conditions focuses on the retrospective analysis of ED visits. It does not allow, however, for any general assumptions to be made on the treatment and diagnostics received by individual patients. Fourth, our data did not allow to control for the day of the week on which ED visits took place or for the triage-rating of patients in ED. Our analyses included only patients with SHI in Germany. ED cases of certain hospitals in North Rhine-Westphalia are not included in the dataset.^36^ Lastly, because we used aggregated data in our regression analyses, our findings are potentially subject to ecological fallacy.^37^ Relationships found at a regional level do not necessarily reflect those at an individual level. Future research using individual data, and/or smaller geographic units, as well as on the transferability of our results to different healthcare settings, would be desirable.

## Supplementary Material

ckz081_Supplementary_Data
