# Supplementary material for: Ambulatory care-sensitive emergency department cases: a mixed methods approach to systemize and analyze cases in Germany
Source: Eur J Public Health. 2019 May 14;29(6):1024–30. doi: 10.1093/eurpub/ckz081 (PMC6896970; doi:10.1093/eurpub/ckz081)
Supplement: ckz081_Supplementary_Data [file ckz081_supplementary_data.docx]

**Supplementary material**

Table A1: Descriptive results

| Variable | N | Mean | Standard Deviation | Min | Max |
| --- | --- | --- | --- | --- | --- |
| AC-sensitive emergency case rate | 338 | 41.03 | 14.19 | 2.00 | 79.76 |
| AC-sensitive emergency case rate -  General Medicine | 338 | 22.77 | 8.99 | 1.45 | 52.13 |
| AC-sensitive emergency case rate - Injuries | 338 | 22.83 | 7.76 | 1.08 | 41.58 |
| Charlson Index | 338 | 1279.28 | 257.54 | 642.81 | 2395.19 |
| AC cases | 338 | 6880.86 | 552.43 | 5446.20 | 8816.23 |
| Income | 338 | 1742.40 | 223.36 | 1345.10 | 345.70 |
| Voter turnout | 338 | 69.56 | 4.35 | 57.40 | 78.5 |
| Distance to hospitals | 338 | 10.29 | 5.14 | 0.00 | 22.00 |
| Physician density | 338 | 170.3 | 61.73 | 78.40 | 410.90 |
| Percentage of non-German citizen | 338 | 7.29 | 4.49 | 1 | 24 |

Table A2: Regression results: individual German states

|  | AC-sensitive emergency case | | AC-sensitive emergency case -  General Medicine | | AC-sensitive emergency case - Injuries | |
| --- | --- | --- | --- | --- | --- | --- |
|  | OLS | SEM | OLS | SEM | OLS | SEM |
| Hamburg | -19.28^+^ | -16.91^+^ | -14.33^*^ | -13.88^*^ | -8.173 | -6.153 |
|  | (10.12) | (9.606) | (6.692) | (6.468) | (5.572) | (5.123) |
| Lower Saxony | 17.07^***^ | 18.36^***^ | 9.126^***^ | 9.337^***^ | 9.454^***^ | 10.94^***^ |
|  | (2.914) | (3.689) | (1.927) | (2.069) | (1.605) | (2.430) |
| North Rhine Westphalia | 23.99^***^ | 24.03^***^ | 11.30^***^ | 11.35^***^ | 15.13^***^ | 14.74^***^ |
|  | (3.086) | (3.800) | (2.042) | (2.162) | (1.700) | (2.510) |
| Baden-Wurttemberg | 14.12^***^ | 14.99^***^ | 4.998^*^ | 5.260^*^ | 10.05^***^ | 10.46^***^ |
|  | (3.363) | (4.069) | (2.225) | (2.358) | (1.852) | (2.623) |
| Bavaria | 11.73^***^ | 12.40^***^ | 3.176^+^ | 3.297^+^ | 9.289^***^ | 9.965^***^ |
|  | (2.842) | (3.552) | (1.880) | (2.004) | (1.565) | (2.352) |
| Saarland | 22.68^***^ | 22.97^***^ | 11.19^***^ | 11.24^***^ | 13.11^***^ | 13.29^**^ |
|  | (4.741) | (6.306) | (3.136) | (3.412) | (2.611) | (4.448) |
| Berlin | 29.21^**^ | 26.21^**^ | 20.96^**^ | 20.04^**^ | 13.37^*^ | 11.53^*^ |
|  | (10.16) | (10.05) | (6.720) | (6.628) | (5.595) | (5.596) |
| Brandenburg | -15.37^***^ | -14.85^***^ | -6.347^**^ | -6.329^*^ | -9.586^***^ | -8.275^**^ |
|  | (3.561) | (4.390) | (2.356) | (2.496) | (1.961) | (2.908) |
| Mecklenburg-Vorpommern | 9.776^*^ | 10.28^*^ | 8.238^**^ | 8.291^**^ | 3.141 | 3.570 |
|  | (4.479) | (5.214) | (2.963) | (3.090) | (2.467) | (3.180) |
| Saxony | 24.15^***^ | 25.29^***^ | 15.83^***^ | 15.88^***^ | 11.69^***^ | 13.58^***^ |
|  | (3.824) | (4.636) | (2.529) | (2.660) | (2.106) | (3.016) |
| Saxony-Anhalt | 16.15^***^ | 17.06^***^ | 11.85^***^ | 11.86^***^ | 6.395^**^ | 8.184^**^ |
|  | (4.032) | (4.694) | (2.667) | (2.765) | (2.221) | (2.989) |
| Thuringia | 13.15^***^ | 12.51^**^ | 6.729^**^ | 6.554^**^ | 7.517^***^ | 7.105^**^ |
|  | (3.457) | (4.158) | (2.287) | (2.410) | (1.904) | (2.666) |

Figure A1: Examples of the Delphi Questionnaire
